# *Tisochrysis lutea* as a Substrate for Lactic Acid Fermentation: Biochemical Composition, Digestibility, and Functional Properties

**DOI:** 10.3390/foods12061128

**Published:** 2023-03-07

**Authors:** Caterina Pagnini, Giacomo Sampietro, Gaia Santini, Natascia Biondi, Liliana Rodolfi

**Affiliations:** Department of Agriculture, Food, Environment and Forestry (DAGRI), University of Florence, Piazzale delle Cascine 18, 50144 Florence, Italy

**Keywords:** *Tisochrysis lutea*, *Lactiplantibacillus plantarum*, fucoxanthin, fermentation, lactic acid, *in vitro* digestibility, radical scavenging activity

## Abstract

Microalgae, because of their high nutritional value and bioactive molecule content, are interesting candidates for functional foods, including fermented foods, in which the beneficial effects of probiotic bacteria combine with those of biomolecules lying in microalgal biomass. The aim of this work was to evaluate the potential of *Tisochrysis lutea* F&M-M36 as a substrate for *Lactiplantibacillus plantarum* ATCC 8014 and to verify fermentation effects on functionality. Bacterium selection among three lactobacilli was based on growth and resistance to *in vitro* digestion. Microalgal raw biomass and its digested residue were fermented in two matrixes, water and diluted organic medium, and analysed for biochemical composition and antioxidant activity along with their unfermented counterparts. Bacterial survivability to digestion and raw biomass digestibility after fermentation were also evaluated. Fucoxanthin was strongly reduced (>90%) in post-digestion residue, suggesting high bioavailability. Raw biomass in diluted organic medium gave the highest bacterial growth (8.5 logCFU mL^−1^) and organic acid production (5 mg L^−1^), while bacterial survivability to digestion (<3%) did not improve. After fermentation, the antioxidant activity of lipophilic extracts increased (>90%). Fermentation appears an interesting process to obtain *T. lutea*-based functional foods, although further investigations are needed to optimize bacterial growth and fully evaluate its effects on functionality and organoleptic features.

## 1. Introduction

Microalgae (including cyanobacteria) are a highly diverse collection of microorganisms [1] that consist of approximately 50,000 species distributed in all environments [2]. Their potential as food is due to their balanced biochemical composition and high nutritional value [3,4]. In fact, microalgae are rich (about 40–50% and, in some species, up to 70% of biomass dry weight) in high-quality proteins, polyunsaturated fatty acids, and bioactive molecules with health-promoting properties [5], which make them strong candidates for the production of nutraceuticals and functional foods [1]. In addition, microalgae are potentially sustainable resources, as their production does not require fertile land or pesticides; they are efficient in the use of nutrients, thus reducing the risk of water body pollution with unused fertilizers; and they can be grown in non-potable water as well as in brackish and seawater [6,7,8]. Nowadays, the global market for dietary supplements is dominated by *Arthrospira* and *Chlorella* (about 15,000 and 5000 t of dry biomass annually, respectively [9]), which is also due to their long history of human consumption, which allows them to be considered safe by novel food regulations around the world [10,11]. In the food industry, the current trend is to incorporate microalgal biomass or microalgae-derived compounds (e.g., pigments) as ingredients in food formulations [12].

The use of microalgae in the food industry includes the development of functional fermented foods. Fermentation allows the addition of the beneficial effects of probiotic bacteria to the useful biomolecules present in the microalgal biomass and may improve organoleptic features [13]. Fermentation has been investigated on several microalgae, primarily *Arthrospira* [14], with variable results. *Arthrospira platensis* (*A. platensis*) F&M-C256 was shown to be a suitable substrate for *Lactiplantibacillus plantarum* (*L. plantarum*) ATCC 8014 growth, while the fermentation process improved antioxidant capacity and phenolic content [15,16]. Martelli et al. [17] observed an increase in the concentration of lactic acid bacteria grown on a commercial *A. platensis* biomass depending on the microalgal biomass initial concentration, the composition of the bacterial mixed population, and the substrate used. A commercial *A. platensis* biomass proved to be a suitable substrate for solid-state fermentation with *Lacticaseibacillus casei* (*L. casei*) 2240 and *Lacticaseibacillus rhamnosus* (*L. rhamnosus*) GG, which also led to an improvement in organoleptic characteristics [18]. Kaga et al. [2] obtained low fermentation performances by *L. plantarum* Urama-SU4 and *Lactobacillus lactis* Urama-SU1 with several microalgal biomasses (including *A*. *platensis*) suspended in water, while better results were achieved with biomasses from a wild-collected *Nostoc commune* (*N. commune*) and from *Euglena* sp. grown on sake lees. The addition of a commercial *Euglena gracilis* biomass improved the growth of *Faecalibacterium prausnitzii* JCM31915 [19], while the addition of Klamath *Aphanizomenon flos-aquae* (*A. flos-aquae*) biomass at 6% increased the growth of *Lactobacillus acidophilus* (*L. acidophilus*) DDS [20]. 

The marine haptophyte *Tisochrysis lutea* (*T. lutea*) is a unicellular biflagellate species covered by several layers of scales [21]. *T. lutea* is widely used in aquaculture [22,23] since it is a valid source of docosahexaenoic acid (DHA) [24], an omega-3 long-chain polyunsaturated fatty acid constituting an important component of cell membranes, which is considered to play a role in the prevention of cardiovascular diseases and probably of neurological disorders [24]. Besides DHA, it also contains other bioactive compounds, such as fucoxanthin [25,26] and phenols [27,28], together with a high amount of proteins and fibres [26]. The beta-glucan chrysolaminarin is the storage product [29]. Despite its valuable biochemical profile, *T. lutea* is not much investigated in the functional food industry, and it is not currently approved for use in food; nevertheless, several studies show its safety as a dietary product. Nuno et al. [30] observed no acute toxicity in rats fed with *T. lutea* at a dosage of 50 mg day^−1^; in addition, after 8 weeks, *T. lutea* supplementation promoted body weight loss in healthy rats and maintenance in rats with diabetes. Niccolai et al. [10] found a rather high (6 g of extracted dry biomass L^−1^) IC_50_ in human fibroblasts and in an *Artemia salina* assay for *T. lutea* F&M-M36 methanolic extracts. Bigagli et al. [31], in an *in vivo* study with rats fed a diet enriched with *T. lutea* (equivalent to 159 g of biomass day^−1^ in a 70 kg man), observed no changes in growth or behaviour; in addition, *T. lutea* exhibited ipolipidemic effects. 

The objectives of the present work were to evaluate the potential of *T. lutea* F&M-M36 lyophilized biomass as a substrate for *L. plantarum* ATCC 8014 (selected among three lactic acid bacteria) growth, as well as to investigate the role played by the indigestible fraction of the microalgal biomass in bacterial growth (prebiotic effect). Finally, we aimed to verify the effect of fermentation on functional properties (the presence of functional components such as pigments and radical scavenging activity) as well as to evaluate whether fermented microalgal biomass could exert a protective role towards the probiotic bacterium during a simulated digestive process.

## 2. Materials and Methods

### 2.1. Microorganisms

The marine haptophyte *T. lutea* F&M-M36 belongs to the Fotosintetica & Microbiologica Culture Collection (Florence, Italy), and the lyophilized biomass, obtained by cultivating the microalga in Green Wall Panel (GWP^®^) photobioreactors [32] under natural light, was purchased from Archimede Ricerche S.r.l. (Camporosso, Imperia, Italy). The biomass was stored at −21 °C until use.

The lactic acid bacteria (LAB) used were *L. plantarum* ATCC 8014, *Lactobacillus delbrueckii* subsp. *bulgaricus* (*L. bulgaricus*) LB28A, and *L. casei* LB28B. *L. plantarum* ATCC 8014 was purchased from Cruinn Diagnostic Ltd. (Dublin, Ireland). *L. bulgaricus* LB28A and *L. casei* LB28B were provided internally and were originally isolated from fermented products. Cultures were maintained in de Man, Rogosa, Sharpe (MRS) agarised medium (Oxoid Ltd., Basingstoke, United Kingdom). 

### 2.2. Experimental Plan

**Figure 1:**
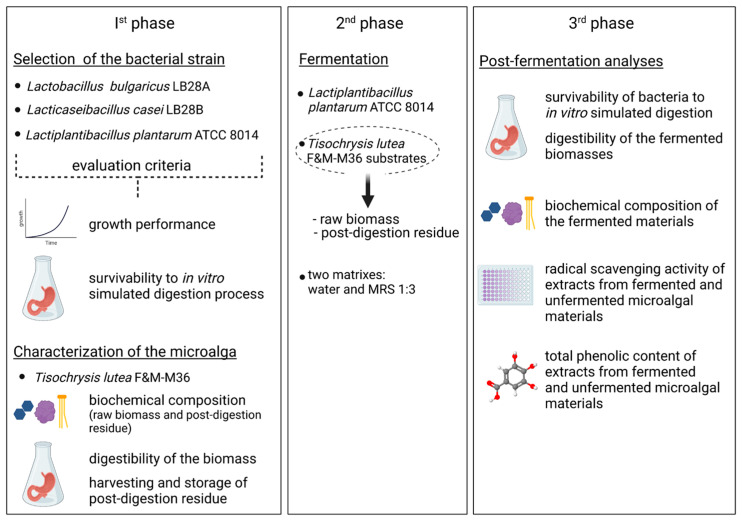
Outline of the experimental design of the work.

This study was organized in three phases (Figure 1): the first comprised the characterization of *T. lutea* F&M-M36 raw biomass and the choice of LAB strain to be used in the fermentation trial, which was carried out in the second phase, while the third included characterization of the fermented materials (raw biomass and post-digestion residue).

More specifically, *T. lutea* F&M-M36 raw biomass was characterized for its biochemical profile and for *in vitro* digestibility. The dried digested residue was stored for further use. Concerning bacteria, three LAB strains were characterized for their growth performance and for resistance to *in vitro* simulated digestion, expressed as cell survivability. Once *L. plantarum* ATCC 8014 was selected among the bacteria, a fermentation trial was set up with *T. lutea* F&M-M36 raw biomass and post-digestion residue. The dried products obtained at the end of fermentation were analysed for biochemical composition, digestibility, and radical scavenging activity and compared with the unfermented counterparts. Survivability of *L. plantarum* ATCC 8014 to *in vitro* simulated digestion was evaluated also after fermentation with *T. lutea* F&M-M36 biomass.

### 2.3. Characterization of T. lutea F&M-M36 Unfermented and Fermented Materials

#### 2.3.1. Biochemical Composition

Total protein was estimated following Lowry et al. [33] through calibration with bovine serum albumin (Sigma Aldrich, Milan, Italy). Carbohydrates were determined by the phenol-sulphuric acid method [34] and calibration with glucose (Sigma Aldrich). Lipids were analysed by carbonization with sulphuric acid [35] after extraction with chloroform:methanol (CHCl_3_:MeOH)1:2, phase separation followed by solvent evaporation [36] and calibration with tripalmitin (Sigma Aldrich). Ashes were determined by mineralizing preweighed aliquots of biomass in a muffle furnace at 500 °C for 24 h. Total dietary fibres (TDF) were estimated using an enzyme kit (K-TDFR-100A, Megazyme, Bray, Ireland) following AOAC 985.29 method.

Fucoxanthin content was determined as follows: 15 mg of each sample was added with 270 μL of Sudan Red (Sigma Aldrich) solution (1 mg mL^−1^ in MeOH/methyl tertiary butyl ether (MTBE) 4:1 solution), monitoring standard for UV–Vis lamp, 150 μL of β-apo-carotenal (Sigma Aldrich) solution (1 mg mL^−1^ in MTBE), internal standard for quantification, and 7.5 mL of pure MeOH. The suspensions were heated at 60 °C for 15 min, at the end of which, they were vortexed and added with 7.5 mL of diethyl ether/petroleum ether solution (50:50) and 5 mL of NaCl solution (20% in water). The suspensions were vortexed again to allow phase separation, and the upper phase was collected. Steps starting with addition of diethyl ether/petroleum ether solution were repeated twice. The upper phases were collected in a rotary evaporation flask, dried under vacuum (Rotavapor RII, Büchi, Flawil, Switzerland), and resuspended in 3 mL of MeOH/MTBE 4:1 solution. The extracts were analysed by HPLC (1050, Hewlett Packard, Palo Alto, CA, USA) equipped with C30 reverse phase column (YCM Carotenoid, 4.6 mm × 250 mm, 5 μm particle size) (Waters, Millford, MA, USA) and UV photodiode array detector (Hewlett Packard 1050, USA) at 25 °C. A gradient method was adopted: 100% eluent A (81% MTBE, 10% MeOH, 9% deionised water) for 1 min, passing to 92% eluent A and 8% eluent B (93% MTBE, 7% MeOH) in 7 min, then to 10% eluent A and 90% eluent B in 8 min 50 s, holding this ratio for 2 min 20 s, then going back to 100% eluent A in 50 s, and holding this condition for further 12 min. The injection volume was 20 μL with a constant flow rate of 1 mL min^−1^. Detection was held at 450 nm. 

An aliquot of the extracts obtained for fucoxanthin determination was diluted 1:60 in methanol and read spectrophotometrically (Cary 60, Agilent, Santa Clara, CA, USA) at 470, 652, and 750 to determine total carotenoids according to the equations for pure MeOH of Lichtenthaler and Buschmann [37].

#### 2.3.2. In Vitro Digestibility of Microalgal Raw Biomass

*In vitro* digestibility of *T. lutea* F&M-M36 raw biomasses (before and after fermentation) was evaluated following Boisen and Fernández’s [38] method modified by Niccolai et al. [26], reproducing digestion occurring in the proximal tract (stomach and duodenum) of monogastric animals. Triplicate samples (1 g) (particle size ≤1 mm) were weighed in 100 mL flasks. Concurrently, 3 flasks for blanks were set up. Phosphate buffer (25 mL/sample, 0.1 M, pH 6.0) and HCl (10 mL/sample, 0.2 M, pH 2) were sequentially added to each flask, and then pH was adjusted to 2.0 by 5 M HCl addition. Then, 2 mL of porcine pepsin (0.8 FIP-U mg^−1^, Applichem, Darmstadt, Germany) solution (10 mg mL^−1^ in H₂O) was added to each flask. Flasks were incubated for 6 h at 39 °C under constant agitation (150 rpm). After incubation, phosphate buffer (10 mL/sample, 0.2 M, pH 6.8) and NaOH (5 mL/sample, 0.6 M) were sequentially added to each flask, and pH was adjusted to 6.8 with 5 M NaOH. After, 10 mL of a porcine pancreatin (42,362 FIP-U g^−1^, Applichem) solution (50 mg mL^−1^ in 1:1 ethanol-EtOH:water) was added, and then the flasks were incubated for 18 h at 39 °C under constant agitation (150 rpm). At the end, flask content was centrifuged (2840 g, with 10 min cycles; NEYA 8, REMI, Mumbai, India) in previously tared tubes. Once the supernatants appeared limpid, they were discarded, and pellets were washed with H₂O to remove salts and centrifuged again (2840 g for 30 min). Then, the pellets in the tubes were dried in an oven at 50 °C until constant weight. Digestibility was calculated as the difference between initial and final (after blank value subtraction) biomass weights divided by the initial biomass weight.

### 2.4. Characterization of Bacterial Strains

#### 2.4.1. Bacterial Growth

Bacterial growth was determined in MRS broth. Cultures were held in 250 mL flasks and maintained under constant agitation (150 rpm) at 28–30 °C, starting by diluting an actively growing inoculum culture. Bacterial concentration was measured as optical density (OD) at 600 nm. At the same time, the total number of cells was counted by a Neubauer chamber with 0.01 mm depth (Marienfeld, Lauda-Königshofen, Germany). Growth rate was calculated as the difference between OD natural logarithm at two different times divided by the time interval.

#### 2.4.2. Bacterial Survivability to *In Vitro* Digestion

To determine bacterial survivability in a monogastric digestive system, the protocol described by Naissinger da Silva et al. [39], simulating the transit through stomach and duodenum, was used and modified as follows. 

Biomass concentration (dry weight) of inoculum cultures was determined by filtering 2 mL of each culture on preweighed mixed cellulose ester membrane filters (Test Scientific, Perugia, Italy) with 0.22 μm pore size. Membranes were dried at 105 °C until constant weight. Dry biomass concentration (g L^−1^) was calculated as the difference between postfiltration dry weight and prefiltration (empty filter) dry weight, and the resulting value was divided by filtered volume. Then, culture aliquots corresponding to 0.25 g of dry weight each were centrifuged at 2560× *g* for 8 min. Supernatants were discharged while pellets were suspended in 6.5 mL of Nutrient Broth (Merck KGaA, Darmstadt, Germany) and transferred in 50 mL flasks. Each culture was tested in duplicate, and two controls (blanks) with only reagents were prepared. To each flask, 1.5 mL of porcine pepsin (0.8 FIP-U mg^−1^, Applichem) solution (5 mg mL^−1^ in 0.1 M HCl) was added, and pH was adjusted to 4.6 with 5 M HCl. Flasks were incubated for 20 min at 37 °C under constant agitation (150 rotations per minute, rpm). pH was then adjusted to 2.0 with 5 M HCl, and the flasks were incubated again for 70 min under the same conditions. Samples in two flasks were then adjusted to pH 5.0 with 5 M NaOH and kept for plate counting. The remaining two samples and controls were added with 2.5 mL of porcine pancreatin (42.362 FIP-U g^−1^, Applichem) solution (12.5 mg mL^−1^ in 0.1 M NaHCO₃), pH was adjusted to 5.0 with 5 M NaOH, and further incubation for 20 min was performed under the same conditions. Finally, pH was adjusted to 6.5 with 5 M NaOH prior to the last incubation step of 90 min under the same conditions. At the end, samples for plate counting were taken from each test and control flask. Plate counting was performed by serially diluting samples 1:10 in 96-well plates (Evergreen Scientific, Buffalo, NY, USA). Enumeration was performed by drop count technique [40], plating 10 μL of each dilution in triplicate on MRS medium. Colony forming units (CFU) were counted under a microscope (Eclipse 50i, Nikon, Tokio, Japan) after incubation at 37 °C for 24–48 h. Survivability was expressed as percentage compared to counts at the start of the process (just after suspension of the cell pellet in Nutrient Broth). The remaining culture volumes were centrifuged (2560× *g* for 10 min), supernatants were discharged, while pellets, after washing with deionized water, were dried at 50 °C until constant weight to determine digestibility of bacterial biomass.

### 2.5. Fermentation Trial

**Figure 2:**
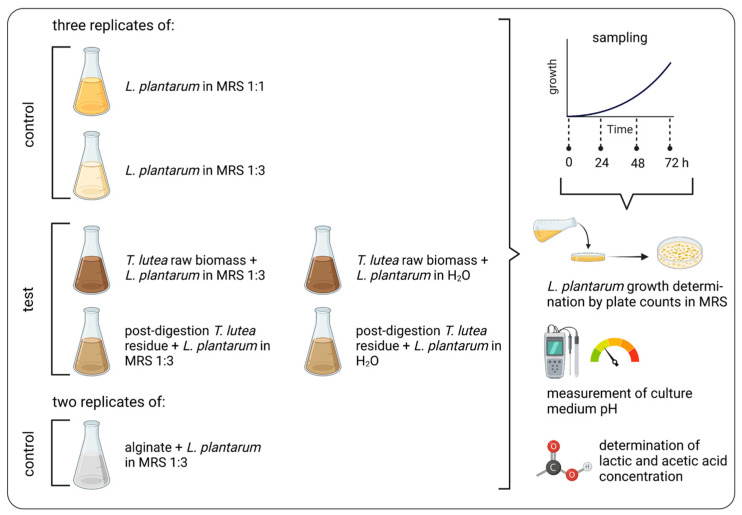
Outline of the fermentation trial of *Tisochrysis lutea* F&M-M36 raw biomass and post-digestion residue with *Lactiplantibacillus plantarum* ATCC 8014. Fermentation parameters followed during the trial are also reported.

*L. plantarum* ATCC 8014 was selected for the fermentation trial with *T. lutea* F&M-M36 raw biomass post-digestion residue. The fermentation trial was set up and conducted as reported in Figure 2. Two controls were prepared with *L. plantarum* in standard MRS (hereafter named 1:1) and in MRS with one-third of the dose indicated in the recipe (hereafter named 1:3). The investigated conditions were *L. plantarum* + *T. lutea* raw biomass suspended in H₂O, *L. plantarum* + *T. lutea* raw biomass suspended in MRS 1:3, *L. plantarum* + *T. lutea* post-digestion residue suspended in H₂O, and *L. plantarum* + *T. lutea* post-digestion residue suspended in MRS 1:3. All the mentioned treatments and controls were set up in triplicate. A control for prebiotic activity was also prepared in duplicate: *L. plantarum* + sodium alginate dissolved in MRS 1:3. All the treatments and the controls were sampled just after inoculation (T₀) after 24 (T₂₄), 48 (T₄₈), and 72 (T₇₂) h from the start of fermentation. 

Prior to the fermentation trial, the bacterial culture was kept in active growth phase, and for inoculation, the total number of cells was estimated by counting with a Neubauer chamber. On the basis of cell count, 30 mL of inoculum culture at 10^8^ cell mL^−1^ were centrifuged for each treatment/control to be set up. Pellets were resuspended in 30 mL of fermentation matrix (MRS 1:1, MRS 1:3 or H₂O) in 50 mL flasks. Then, 2.5 g of raw microalgal biomass or microalgal post-digestion residue was added to the corresponding flask. A total of 0.5 g of sodium alginate was added to each positive control flask. The microalgal biomasses, sodium alginate and the enzyme powders were not sterile, while all the other materials and solutions were sterile.

To evaluate the fermentation process development, bacterial growth was determined at each sampling by drop plating as described in §2.4.2. At each sampling, pH (pH 510, XS Instruments, Carpi, Modena, Italy) of the culture medium after centrifugation (see below for conditions) was measured. For determination of organic acids concentrations, 4 mL aliquots were collected at each sampling and centrifuged (2840× *g* for 20 min). Supernatants were collected and stored at −21 °C until analysis. Samples were sent to FoodMicro Team S.r.l., spin-off of the University of Florence, for analysis. L-lactate and acetic acid were determined by enzymatic assays [41,42] through an Hyperlab Plus analyzer (Steroglass S.r.l., Perugia, Italy). To obtain net acetate production, acetate concentration present in the medium (5 g L^−1^ of trihydrate sodium acetate in MRS 1:1, reduced to one-third in MRS 1:3) was subtracted from the acetate concentration obtained from supernatant analysis.

At the end of fermentation, cultures with raw *T. lutea* biomass were tested in duplicate for digestibility: 4.5 mL of an equal mixture of the three replicates was centrifuged (2560× *g* for 8 min) and resuspended in phosphate buffer. A blank was also set up in duplicate. Digestibility was determined following the protocol used for microalgal biomass (§2.3.2). Bacterial survivability was determined after treatment with pepsin and after pancreatin (end of the process) as previously described. 

### 2.6. Determination of Extracts Radical Scavenging Activity, Pigment and Total Phenolic Content

Unfermented and fermented raw biomasses and digested residues were characterized for radical scavenging activity (RSA) through DPPH assay [43]. Lyophilized material (about 0.5 g) was extracted using, in succession, three solvents with increasing polarity: hexane, CHCl_3_:MeOH 1:2, and 30% EtOH in water. Each extraction lasted 8 h. After extraction, solvent was separated by filtration on paper, and then residual solvent was allowed to evaporate from the material before addition of the successive solvent. Separated solvents were evaporated under vacuum, and residues were suspended in 5 mL of methanol (for hexane and CHCl_3_:MeOH 1:2) or 30% EtOH. Dry weight of the extracts was determined by absorbing a known volume of each extract on preweighed glass fibre filters (Filter Lab, Barcelona, Spain) and then dried at 50 °C until constant weight.

Dilutions of the extracts were prepared so as to obtain final solutions of 1 and 2 g L^−1^ (extract dry weight). These dilutions were tested for RSA: 0.5 mL of each extract was added with 0.5 mL of a 3·10^−4^ M 2,2-diphenyl-1-picrylhydrazyl (DPPH) (Sigma Aldrich) solution in dimethyl sulfoxide (DMSO) [43], allowed to react in the dark for 30 min, and read at 517 nm. The “analysis blank” was prepared with 0.5 mL of DMSO added with 0.5 mL of DPPH solution. A “sample blank” was also prepared to correct for absorption due to pigments; in this case, 0.5 mL of each extract was added with 0.5 mL of DMSO. To calculate RSA, the following equation was used [44]:

$$\mathrm{RSA}\;\left(\%\right)=(A_{S+\mathrm{DPPH}}-A_{S+\mathrm{DMSO}})/A_{b}\times 100$$

where A= absorbance at 517 nm; S= sample; and b= analysis blank. RSA activity was also expressed as vitamin E (Sigma Aldrich) equivalent antioxidant capacity. Vitamin E was dissolved in methanol, and different concentrations in the range of 0–100 mg L^−1^ were analysed as described for the samples to obtain a calibration curve.

Pigment (chlorophyll *a* and *c* and total carotenoids) content in the extracts was determined with spectrophotometric analysis. Extracts were diluted (33 to 200 folds) in pure MeOH, and equations for pure MeOH were used to calculate chlorophyll [45] and total carotenoid [37] concentration. Pigment content was then calculated as mg g^−1^ of dry extract based on the extract dry weight previously determined.

Total phenolic content was determined only for CHCl_3_:MeOH 1:2 and 30% EtOH extracts and diluted to have a dry weight of 2 g L^−1^. For each extract, 100 μL were added with 2% Na_2_CO_3_ solution in water (2 mL), then, after 2 min, with 100 μL of 1 N Folin Ciocalteu reagent (Sigma Aldrich). The samples were allowed to react in the dark for 30 min and then read at 720 nm. Total phenolic content was calculated as gallic acid equivalents per unit of extract dry weight based on a calibration curve prepared with gallic acid (Sigma Aldrich) (0–300 mg L^−1^).

### 2.7. Statistical Analysis

Data were analysed by means of Student’s t-test or one-way analysis of variance (ANOVA) followed by Tukey’s or Dunnett’s multicomparison test performed using Prism 6 (GraphPad Software, Boston, MA, USA). The level of significance was *p* < 0.05.

## 3. Results

### 3.1. Selection of Bacterial Strain

The growth rates of the three bacterial strains tested are shown in Table 1. Considering both the OD value and cell counts by counting chamber, *L. plantarum* showed the highest and *L. casei* the lowest growth rate.

**Figure 3:**
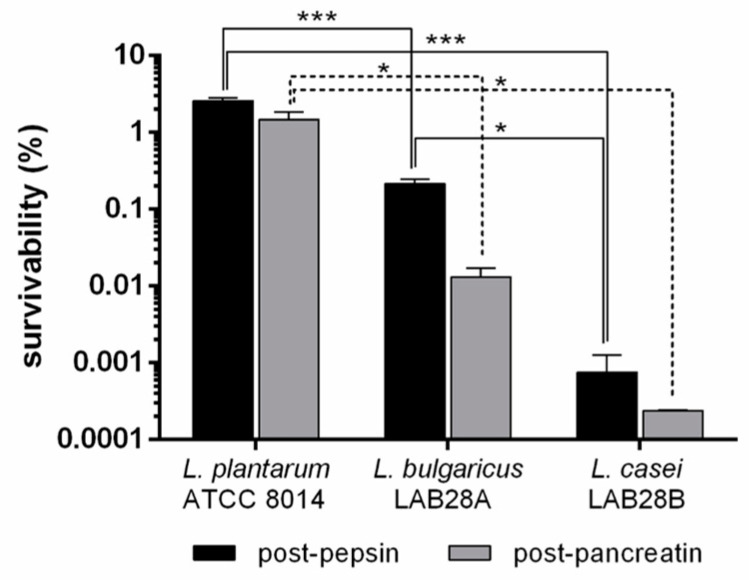
Bacterial survivability to digestion process. Survivability, expressed as percentage with respect to initial cell concentration, of three lactic acid bacteria to *in vitro* digestion after treatment with pepsin alone (post-pepsin) and with pepsin followed by pancreatin (post-pancreatin). Calculations are based on CFU counts. *** highly significant differences (*p* < 0.001); * significant differences (*p* < 0.05).

Bacterial survivability to *in vitro* digestion is reported in Figure 3 and is expressed as a percentage of the value at the start of the trial based on CFU mL^−1^ counts. In the case of *L. casei*, the number of CFU was strongly reduced after treatment with pepsin, resulting in a survivability of <0.01%, which further decreased after treatment with pancreatin (<0.001%). *L. plantarum* showed the best survivability, equal to 2.6% after treatment with pepsin and 1.5% after that with pancreatin. *L. bulgaricus* showed intermediate survivability (0.3% and 0.01% after pepsin and pancreatin, respectively). After pepsin, the differences were significant (*p* < 0.05) among all strains, while after pancreatin, the survivability of *L. plantarum* was significantly higher compared to that of the other two strains, which showed no significant difference between them.

### 3.2. Fermentation

**Figure 4:**
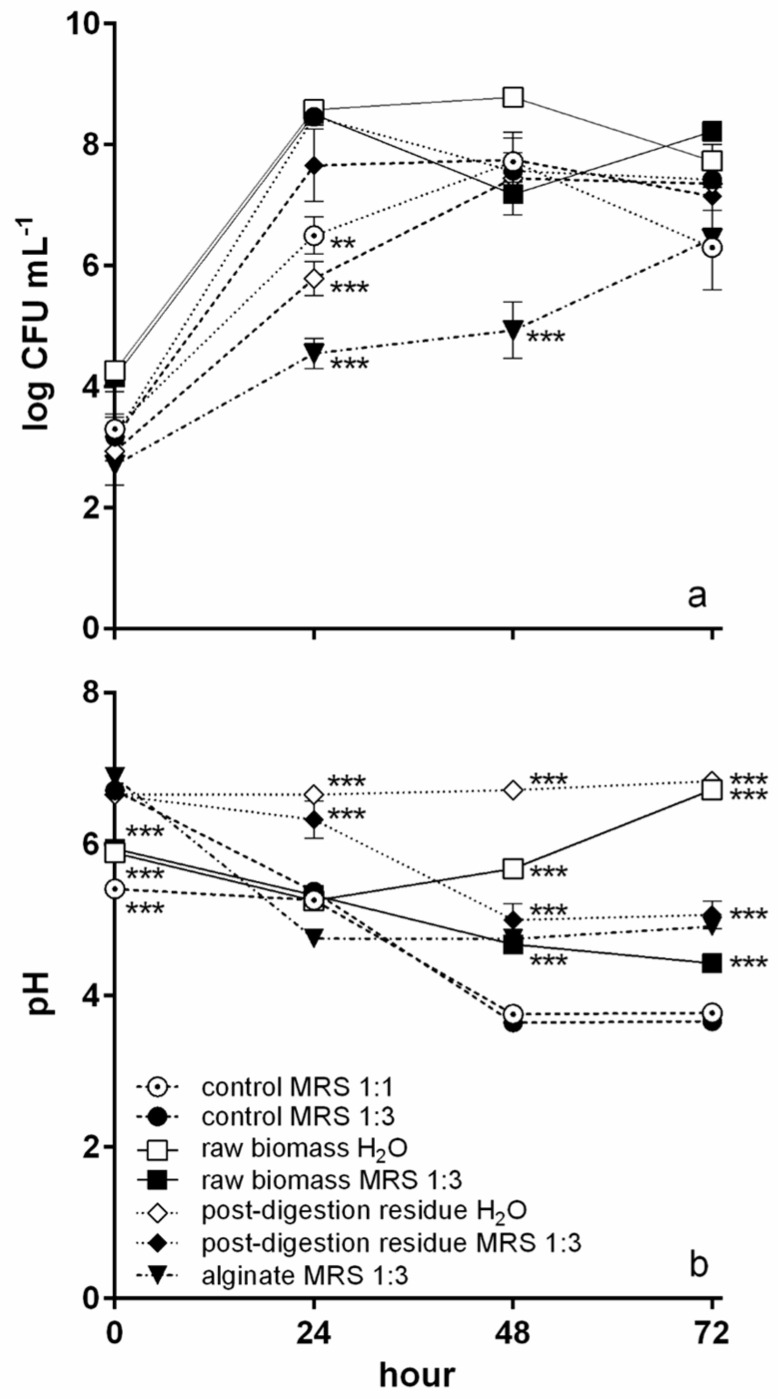
Fermentation trial. (**a**) *L. plantarum* ATCC 8014 growth curves and (**b**) pH variation during fermentation in the controls (MRS 1:1, MRS 1:3), in the positive control (alginate in MRS 1:3), and in cultures with *T. lutea* F&M-M36 raw biomass and post-digestion residue in the two matrixes (H_2_O and MRS 1:3). Data are expressed as mean ± standard error. ** significant difference (*p* < 0.01); *** highly significant difference (*p* < 0.001) compared to values of MRS 1:3 control.

*L. plantarum* was chosen for fermentation as it was the most resistant to digestion. *L. plantarum* growth curves during fermentation in the different conditions tested are shown in Figure 4a. In MRS 1:1, *L. plantarum* grew up to 7.7 logCFU mL^−1^ after 48 h, and then the concentration decreased. In MRS 1:3, the peak of growth was reached after 24 h (8.5 logCFU mL^−1^). In the presence of *T. lutea* raw biomass, the growth dynamic changed according to the fermentation matrix. In H₂O, the *L. plantarum* concentration increased until 48 h, reaching a value of 8.8 logCFU mL^−1^, while in MRS 1:3, the growth was biphasic with the highest concentration value reached after 24 h (8.5 logCFU mL^−1^) and a new increase (8.2 logCFU mL^−1^) after 72 h. The *T. lutea* post-digestion residue led to a lower *L. plantarum* growth, reaching its maximum after 48 h (7.4 and 7.8 logCFU mL^−1^ in H₂O and MRS 1:3, respectively). The control with sodium alginate dissolved in MRS 1:3 showed the lowest *L. plantarum* growth, which reached its maximum after 72 h (6.4 log CFU mL^−1^). *L. plantarum* growth was significantly lower compared to the control in MRS 1:3 only after 24 h in the MRS 1:1 control, in the culture with post-digestion residue suspended in H_2_O, and in that with alginate, which was the only curve to be significantly lower also after 48 h.

Curves of pH during fermentation are shown in Figure 4b. In the MRS 1:1 control, the pH decreased from 5.4 to a minimum of 3.7 after 48 h, remaining stable until the end of the trial. In the MRS 1:3 control, the pH showed a sharper decrease in the first 24 h, reaching the minimum value after 48 h (from 6.7 to 3.4). With *T. lutea* raw biomass suspended in MRS 1:3, a progressive pH decrease was observed until the end of the trial (minimum value 4.4). With *T. lutea* post-digestion residue suspended in MRS 1:3, the pH was higher and reached its minimum after 48 h (from 6.6 to 5.0). In the presence of sodium alginate, the pH decreased to 4.8 after 24 h remaining constant until the end of the trial. Considering the water matrix, in cultures containing *T. lutea* raw biomass, the pH reached the minimum after 24 h (5.2), then increased until the end of fermentation, whereas with *T. lutea* post-digestion residue, the pH remained constant throughout (about 6.7). The initial pH values were all significantly (*p* < 0.01) lower compared to the MRS 1:3 control except for the cultures containing alginate and *T. lutea* post-digestion residue in H_2_O (Figure 4b). After 24 h, the pH was significantly different (higher) from the MRS 1:3 control only in the cultures with post-digestion residue, while at the remaining sampling times, all pH values except that of the MRS 1:1 control were significantly higher than in the MRS1:3 control. If the fermentation matrix is considered, the pH was significantly different between cultures in H_2_O and MRS 1:3 only after 48 and 72 h (*p* < 0.05) for both substrates.

**Figure 5:**
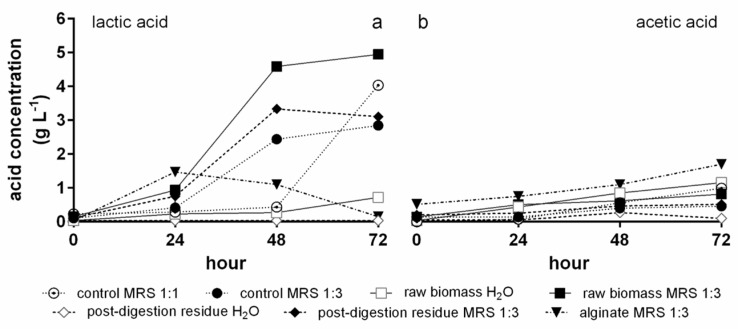
Organic acid production during fermentation. Production of lactic (**a**) and acetic (**b**) acid by *L. plantarum* ATCC 8014 in the controls (MRS 1:1, MRS 1:3), in the alginate control in MRS 1:3, and with *T. lutea* F&M-M36 raw biomass and post-digestion residue in the two matrixes (H_2_O and MRS 1:3). Data are expressed as mean ± standard error.

Lactic and acetic acid concentrations are shown in Figure 5. In MRS 1:1 control lactic acid (Figure 5a) showed a huge increase, reaching a maximum of 4.0 g L^−1^, in the time interval from 48 to 72 h, whereas in MRS 1:3 control the highest increase was observed between 24 and 48 h, although the maximum was attained at the end of the trial (2.8 g L^−1^). A similar behaviour, at higher concentrations (maximum of 4.9 g L^−1^), was observed in the culture with *T. lutea* raw biomass in MRS 1:3, while in the presence of post-digestion residue in MRS 1:3 the curve was similar until 48 h, where the maximum was reached (3.3 g L^−1^). With alginate in MRS 1:3, lactic acid attained the highest value (1.47 g L^−1^) after 24 h. In the cultures with H₂O as the matrix, lactic acid was very low during the whole trial, reaching a maximum of 0.35 g L^−1^ after 48 h with *T. lutea* raw biomass, whereas with postdigestion residue never surpassed the detection limit of the method. 

Acetic acid production (Figure 5b) was, in general, lower than that of lactic acid. Concentrations generally increased from the start to the end of the trial. The highest values were attained with alginate (1.7 g L^−1^), *T. lutea* raw biomass in H₂O (1.2 g L^−1^), and the MRS 1:1 control (1.0 g L^−1^). The only exception was *T. lutea* post-digestion residue in H₂O, in which the maximum acetic acid concentration (0.3 g L^−1^) was reached after 48 h of fermentation.

### 3.3. Fermented Materials

#### 3.3.1. Biochemical Composition

In Table 2, the biochemical composition of *T. lutea* raw biomass fermented in both matrixes is compared with that of unfermented raw biomass. Considering proximate composition, only carbohydrate content resulted as significantly different (higher) in the unfermented compared to the two fermented biomasses. The post-digestion residue had a composition not significantly different from that of the raw biomass (*p* > 0.05). Among fermented residues, the only component showing a significant difference with the unfermented residue was carbohydrates when the fermentation matrix was MRS 1:3. Significant differences were instead present for functional molecules, such as total carotenoids and fucoxanthin, which were strongly affected by digestion (*p* < 0.001) and by fermentation of raw biomass (Table 2), whereas no decrease during fermentation of post-digestion residue was observed. 

**Figure 6:**
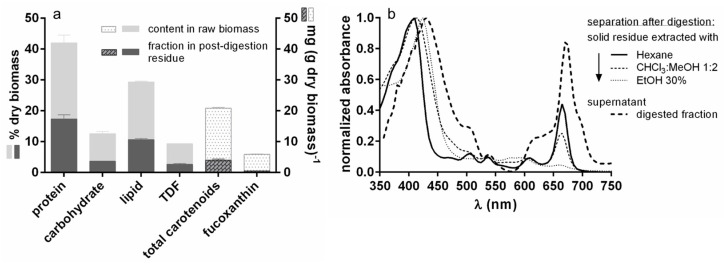
Distribution of raw biomass components in the different digestion fractions. (**a**) Fraction of the components of *T. lutea* F&M-M36 raw biomass remaining in the solid residue after the digestion process. Data are expressed as mean ± standard error. (**b**) Normalized absorbance spectra of the pigments in extracts from the solid post-digestion *T. lutea* F&M-M36 residue and in the supernatant containing the digested fraction.

The fraction of each raw biomass component remaining in the residue at the end of the *in vitro* digestion of *T. lutea* biomass is illustrated in Figure 6a (dark-coloured bar). The less digested component was protein followed by lipids (about 40 and 36% of the content in the initial biomass was present in the residue). The components most strongly reduced during digestion were carbohydrates and TDF (about 28% of the value in the initial biomass present in the residue). Functional components such as total carotenoids and, in particular, fucoxanthin were also highly reduced during digestion (only 19 and 7%, respectively, of the content in initial biomass present in the residue). 

Figure 6b reports the pigments normalized absorbance spectra of post-digestion *T. lutea* solid residue extracted with hexane, CHCl_3_MeOH 1:2, and EtOH 30% and of the digestion supernatant (i.e., the digested fraction). With all three solvents used, the highest absorbances were registered from 400 to 450 nm (chlorophylls and carotenoids) and from 660 to 670 nm (chlorophylls), where the highest value was that of the digestion supernatant. ETOH 30% extract showed high absorbances only in the blue region.

#### 3.3.2. Digestibility of *T. lutea* F&M-M36 Biomass

**Figure 7:**
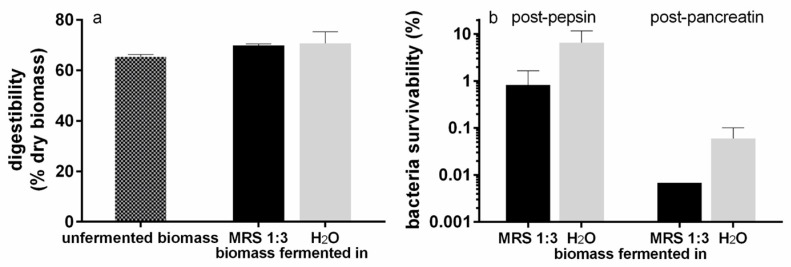
Digestibility of microalgal raw biomass and bacterial survivability. (**a**) Comparison between *T. lutea* F&M-M36 digestibility (% dry biomass) of unfermented biomass and biomass fermented with *L. plantarum* ATCC 8014 suspended in MRS 1:3 or H₂O. (**b**) Survivability of *L. plantarum* ATCC 8014 within the fermented substrate after *in vitro* digestion. Data are expressed as mean ± standard error; statistical significance set at *p* < 0.05.

In Figure 7a, the digestibility (%) of *T. lutea* raw biomass is shown. The microalgal biomass showed a digestibility of about 65%, and no significant differences (*p* > 0.05) were found between the unfermented and the fermented biomasses (independently of the fermentation matrix) or between the two fermentation conditions.

The survivability of *L. plantarum* in the fermented substrate after treatment with the digestive enzymes is shown in Figure 7b. In the substrate fermented in MRS 1:3, the survivability of *L. plantarum* was about 1% after the treatment with pepsin and decreased to lower than 0.01% after that with pancreatin. In the substrate fermented in water, the survivability of *L. plantarum* was around 6.6% after pepsin and about 0.06% after pancreatin. The comparison between the two matrixes showed no significant differences (*p* > 0.05). Furthermore, probably due to the high variability, no significant difference was found within the same matrix after treatment with the two enzymes.

#### 3.3.3. Antioxidant Activity of *T. lutea* F&M-M36 Extracts

**Figure 8:**
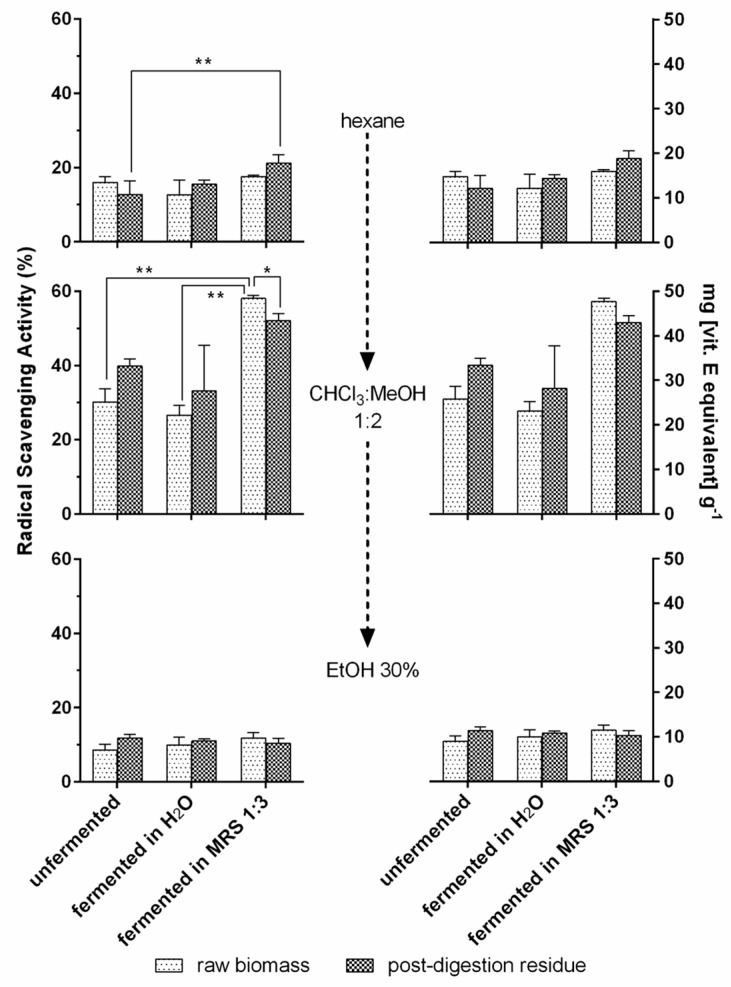
Radical scavenging activity of *T. lutea* F&M-M36 extracts. RSA (%) and the corresponding vitamin E equivalents (mg [Vit. E equivalent] g^−1^) of *T. lutea* F&M-M36 raw biomass and postdigestion residue, unfermented or fermented in H₂O or MRS 1:3, sequentially extracted in hexane, CHCl_3_: MeOH 1:2 and 30% EtOH. Data are expressed as mean ± standard error. ** significant differences (*p* < 0.01); * significant differences (*p* < 0.05).

In Figure 8, the antioxidant activity of sequential extracts obtained from *T. lutea* raw biomass and digested residues is shown. In the first extraction with hexane, a significant difference was detected between the unfermented post-digestion residue (13% RSA) and the post-digestion residue fermented in MRS 1:3 (21% RSA). No significant differences were detected among the other conditions where RSA showed values within the range of 13–18%. The extracts obtained in CHCl_3_:MeOH 1:2 showed the highest RSA values. Raw biomass RSA was significantly higher when fermented in MRS 1:3 (58%) than in H_2_O (27%) and when not fermented (30%). In addition, raw biomass fermented in MRS 1:3 had a significantly higher RSA with respect to the correspondent treatment with post-digestion residue (52%). No other significant differences were detected among the samples. In the last extracts obtained in 30% EtOH, RSA values ranged from 9 to 12%, and no significant differences were detected.

Vitamin E equivalents of RSA values are also reported in Figure 8. The highest equivalents were in the order of 50 mg per gram of extract dry weight and were obtained for raw biomass fermented in MRS 1:3 extracted in CHCl_3_:MeOH 1:2, while the lowest values were in the order of 10 mg g^−1^ and were found for extracts in 30% EtOH.

#### 3.3.4. Pigment Content of *T. lutea* F&M-M36 Extracts

**Figure 9:**
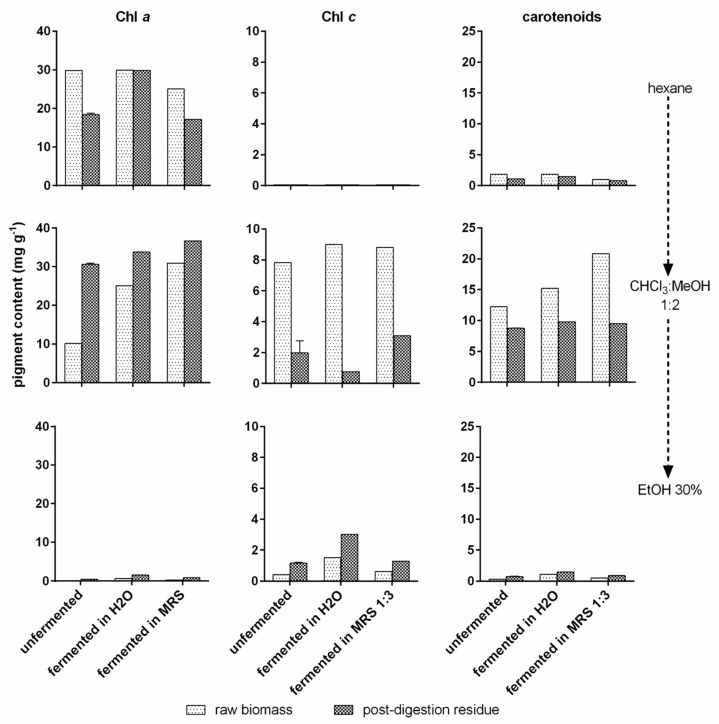
Pigment content in *T. lutea* F&M-M36 extracts. Chlorophyll *a* (Chl *a*), chlorophyll *c* (Chl *c*), and carotenoid content (mg g^−1^) in *T. lutea* F&M-M36 raw biomass and post-digestion residue, unfermented and fermented in H₂O or MRS 1:3, sequentially extracted with hexane, CHCl_3_:MeOH 1:2 and 30% EtOH. Data are expressed as mean ± standard error.

Pigment content (Chl *a*, Chl *c*, and total carotenoids) was quantified (mg g^−1^ of dried extract) in the sequential extracts obtained with hexane, CHCl_3_:MeOH 1:2, and 30% EtOH (Figure 9). Chl *a* was mostly extracted with the first two solvents. In hexane, its content varied little (25–30 mg g^−1^) among the three raw biomass extracts as well as among the three post-digestion residue extracts (30–36 mg g^−1^), although the content was rather higher in post-digestion residue than in raw biomass after fermentation in the organic matrix. In the CHCl_3_:MeOH 1:2 extracts, Chl *a* increased progressively in the raw biomass extracts from unfermented to fermented in water and finally to fermented in an organic matrix, going from 10 to 25 mg g^−1^. In the post-digestion residue, the trend was similar, but the differences were much lower, going from 30 to 37 mg g^−1^. In the extracts with EtOH 30%, Chl *a* content was very low, never exceeding 1.5 mg g^−1^.

Chl *c* was detected only in CHCl_3_:MeOH 1:2 and 30% EtOH extracts, although a very different behaviour was observed between raw biomass and post-digestion residue. In the former, almost all Chl *c* was extracted with CHCl_3_:MeOH 1:2 (7.8–9 mg g^−1^) independently of the treatment. On the contrary, Chl *c* in the post-digestion residues was detected in the two extraction solvents with differences among the treatments: An equal amount was extracted from the unfermented residue (about 1 mg g^−1^); a higher extraction in CHCl_3_:MeOH 1:2 (ca 3 mg g^−1^) was obtained for the residue fermented in MRS 1:3, while the residue fermented in H_2_O showed higher extraction values in 30% EtOH (ca 3 mg g^−1^). 

Finally, for all the treatments, carotenoids were almost exclusively extracted with CHCl_3_:MeOH 1:2. An increasing content (from 12 to 21 mg g^−1^) was observed in raw biomass extracts from unfermented to water-fermented to organic-matrix-fermented biomass. In the post-digestion residue, carotenoid content was lower and similar among the three treatments (9–10 mg g^−1^).

#### 3.3.5. Total Phenolic Content of *T. lutea* F&M-M36 Extracts

**Figure 10:**
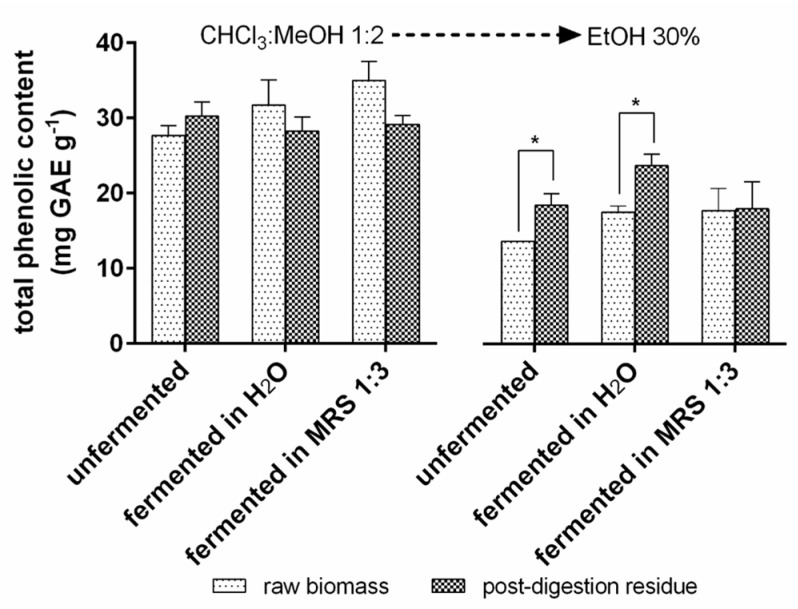
Total phenolic content (mg GAE g^−1^) in the CHCl_3_:MeOH 1:2 and 30% EtOH *T. lutea* F&M-M36 extracts for the two substrates (raw biomass and post-digestion residue) and the different conditions analysed (unfermented, fermented in H₂O, and fermented in MRS 1:3). Data are expressed as mean ± standard error. * significant differences (*p* < 0.05).

Total phenolic content (mg GAE g^−1^) was analysed in CHCl_3_:MeOH 1:2 and 30% EtOH extracts from *T. lutea* raw biomass and post-digestion residue, either unfermented, fermented in H₂O, or in MRS 1:3 (Figure 10). With CHCl_3_:MeOH 1:2, no significant differences were detected, neither between the different substrates (raw biomass or digested residue) nor among the experimental conditions. The total phenolic content in raw biomass was 20.8 mg GAE g^−1^ in the unfermented sample, 30.1 mg GAE g^−1^ in the sample fermented in H₂O, and about 35 mg GAE g^−1^ in the sample fermented in MRS 1:3, while in the post-digestion residue, total phenolic content was 30 mg GAE g^−1^ in the unfermented residue and 28 mg GAE g^−1^ with both fermentation matrixes (H₂O and MRS 1:3).

In the 30% EtOH extract, the total phenolic content of the unfermented raw biomass was significantly lower than in the unfermented post-digestion residue (13 and 18.5 mg GAE g^−1^, respectively). In the samples fermented in H₂O, a significant difference was detected between the two substrates (17 and 23 mg GAE g^−1^ in the raw biomass and post-digestion residue, respectively). No significant differences were detected between the samples fermented in MRS 1:3, where for both substrates, phenolic content was 17 mg GAE g^−1^.

## 4. Discussion

In recent years, increasing attention has been addressed to healthy lifestyles, including nutrition [46,47,48]. Traditional foods, such as fermented products, have also been revisited to improve their healthiness and to better focus on their functionality [13]. Among the improvements, it is worth mentioning the characterization of probiotic properties in bacteria traditionally used to ferment food matrixes, the addition of probiotic strains to the traditional ones, and the investigation of new fermentation substrates [13,49,50]. Microalgae represent an interesting substrate, being endowed with many functional properties and an equilibrated nutritional profile [3,26]. In this framework, a preliminary screening to select the most suitable lactic acid bacterium to perform fermentation with *T. lutea* as the substrate was performed, and the nutritional and functional properties of fermented biomass were compared with those of raw algal biomass.

### 4.1. Probiotic Bacterial Strain Selection

The three bacterial strains tested (*L. plantarum* ATCC 8014, *L. bulgaricus* LAB28A, and *L. casei* LAB28B) showed low survivability to the digestive process simulated *in vitro*. In particular, a larger reduction was observed after the step mimicking the transit through the stomach, where, besides the action of pepsin, the pH is extremely acidic (2.0). The results obtained in the present work (highest survivability of 3% after stomach and 1.5% after intestine passage simulation with *L. plantarum*) are consistent with the literature. A mixed culture of *L. casei*, *L. plantarum*, and *L. rhamnosus* showed a survivability of <1% (approximately from 14 to 11 logCFU mL^−1^) when treated with pepsin at pH 3.0 for 1 h at 38 °C [51]. Naissinger da Silva et al. [39] tested the survivability of commercial probiotic preparations, obtaining in most cases a survivability of 0.2–7.9% after stomach and 1.2–6.3% after stomach plus duodenum simulation. *L. plantarum* PL02 showed a decrease from 8.40 to 5.55 logCFU mL^−1^ (0.14% of survivability) when subjected to the action of acid alone at pH 2.0 for 3 h at 37 °C [52]. Lactobacilli are known to be exopolysaccharide producers [53]. Among the strains tested in this work, *L. casei* LAB28B produced high exopolysaccharide amounts, evidenced by culture medium viscosity. However, exopolysaccharides did not exert a protective effect on cell vital functions against low pH and digestive enzymes, as *L. casei* showed the lowest survivability. On the contrary, if looking at the ability of the enzymes to digest the bacterial cells, exopolysaccharides seem to reduce their accessibility to cell structures, as *L. casei* final digestibility was more than 50% lower than that of the other two bacteria. Since *L. plantarum* ATCC 8014 showed the highest growth rate and resistance to *in vitro* digestion, it was chosen for the fermentation trial. In addition, *L. plantarum* is a versatile lactobacillus, being aerotolerant [54], and is certified as GRAS (generally recognized as safe) and QPS (qualified presumption of safety) [55].

### 4.2. Fermentation of T. lutea F&M-M36 with L. plantarum ATCC 8014

Fermentation was carried out in two different matrixes, water and MRS diluted 1:3. The two matrixes led to similar final bacterial concentrations. Nevertheless, the growth curves showed different patterns: In MRS 1:3 culture, bacterial growth was biphasic, while in water it was monophasic. It is possible that in MRS 1:3, the bacteria already adapted to MRS, first used MRS components, and probably the soluble components of *T. lutea* raw biomass without the need to change their enzyme array; once these readily assimilable compounds were depleted, there was a growth halt during which the bacteria synthesized a proper set of enzymes to use those components of microalgal biomass more resistant to degradation (e.g., β-glucans, proteins, lipids). In water, the bacteria initially grew using the few readily assimilable compounds released from microalgal biomass while, at the same time, adapting to exploit the more difficult ones. Microalgal biomass is a complex substrate composed of large protein, lipid, and complex carbohydrate fractions. It is known that some strains of *L. plantarum* possess proteolytic activity and may also produce enzymes able to degrade complex carbohydrates and lipids [55,56,57]. Moreover, a partial contribution of autochthonous bacteria of *T. lutea* F&M-M36 biomass might be plausible. These bacteria, although present in lower amounts compared to *L. plantarum* (about three orders of magnitude lower, 5.2–5.4 log CFU mL^−1^, as seen in *T. lutea* fermentation without *L. plantarum*), might have contributed to the degradation of the more difficult macromolecules of microalgal biomass, thus providing nutrients useful to *L. plantarum* growth, as already hypothesized in the case of other fermentation substrates [58]. To fully understand these dynamics, further experiments to quantify the different compounds available in the fermentation broth along the process will be necessary. 

The main organic acid produced in the cultures in MRS 1:3 was lactic acid, while in water, acetic acid was produced in similar amounts as lactic acid. The reason for this different behaviour may reside in the complexity of microalgal biomass and its degradation compounds, which might have led also to the formation of different fermentation end-products [59], not characterized in this work, that, in a matrix such as water, might have emerged sooner than in diluted MRS. For example, Taniguchi et al. [60], at the end of a 7-day fermentation of 10% *A. flos-aquae* biomass in water with *L. plantarum* AN7, found, besides a major production of lactic and acetic acid, small amounts of ethanol. *L. plantarum* ATCC 8014 was used to ferment *A. platensis* biomass as the only available substrate in water or in soybean milk providing additional organic compounds, showing similar growth but much different organic acid production patterns [16]. In this work, pH also highlighted the differences between the two matrixes, as it showed a significant negative correlation (*p* < 0.01) with lactic acid production in diluted MRS, while in water, a significant but weaker negative correlation was observed (*p* < 0.05). On the contrary, in neither matrix, the pH significantly (*p* > 0.05) correlated with acetic acid production, although the worse result was again obtained in water. This could be at least partially explained by better preservation of microalgal biomass buffering capacity, a trait observed in several microalgae [61,62], in water compared to MRS 1:3, which has a lower starting pH (about 5.5). The matrix in which fermentation is performed appears, in this work as well as in the literature, of great importance for fermentation outcomes and should be specifically addressed in future experiments. As the matrix impacts fermentative metabolism, it affects organic acid production, an aspect to be dealt with in future developments, considering the high technological relevance of organic acids in food preservation, as antibacterial components and as food taste modifiers [15]. 

*L. plantarum* is a well-known bacteriocin producer with large intraspecific differences [63] that could represent a criterium for strain selection to optimize fermentation. Another criterium for the choice of fermenting bacterium is the response to salinity since *T. lutea* is a marine microalga and its biomass is characterized by rather high salinity values (36 g L^−1^ for *T. lutea* F&M-M36 raw biomass suspended in water at the concentration used in the fermentation trials). The tolerance of *L. plantarum* to increasing salinity has been shown to be strain-dependent with complete growth inhibition at 8–10% salinity [64] and survival rates in excess of 80% at 6% salinity depending on the other culture parameters [65]. It is possible that *L. plantarum* ATCC 8014 did not represent the optimal choice to ferment *T. lutea* biomass, as no screening for salinity tolerance was performed. For future developments, an in-depth investigation to identify the most suitable bacterial strains to ferment marine microalgal biomass will be necessary to optimize the process in terms of growth and functional components.

### 4.3. Potential Prebiotic Effect of T. lutea F&M-M36

To verify whether the growth of *L. plantarum* ATCC 8014 on *T. lutea* F&M-M36 biomass could be due to a prebiotic effect, the residue left after *in vitro*-digestion of biomass was used as a fermentation substrate. Microalgae contain high amounts and varieties of polysaccharides with potential prebiotic effects, such as storage polysaccharides and cell wall components [66]. Several studies have been performed on microalgae prebiotic potential (enhancement of probiotic bacteria growth), mainly for *Arthrospira* and, to a lower extent, *Chlorella* [14,67], although compounds responsible for this type of activity have not been fully elucidated. A prebiotic effect higher than that of a fructooligosaccharide of the digested fraction (the opposite fraction with respect to that used in the present work) of *Chlorella vulgaris* (*C. vulgaris*), *Spirulina platensis* (*S. platensis*), *Desmodesmus maximus,* and *Chlorococcum* cf *hypnosporum* biomasses on human gut microbiota grown anaerobically *in vitro* was found, with the different microalgae stimulating different microbial groups in the microbiota [68]. The starting microalgal biomasses contained high amounts of fibres [68]; however, the amount actually present in the digested biomass was not determined.

In the present work, *L. plantarum* grown with *T. lutea* post-digestion residue showed a lower growth compared to that with raw biomass; moreover, growth was higher in the diluted MRS matrix than in water. Lactobacilli are known to be nutritionally demanding in terms of amino acids, peptides, vitamins, fatty acids, and carbohydrates [58,69], therefore, it is possible that *L. plantarum* growth is unsustainable when the post-digestion residue is used as the only substrate due to a lack of nutritional compounds. However, lactic acid production was higher in *L. plantarum* + *T. lutea* post-digestion residue in MRS 1:3 than in the control culture of *L. plantarum* in MRS 1:3, suggesting that some components within the residue could actually contribute to the fermentative process. Sodium alginate in MRS 1:3 used as a control for prebiotic activity [70], being a component often found in algae, showed the lowest *L. plantarum* growth, producing a lower amount of organic acids compared to both raw biomass and control in MRS 1:3. This indicates a lack of prebiotic activity under the tested conditions. Interestingly, it was the largest producer of lactic acid in the first 24 h, suggesting that not enough nutrients were present for the following period of fermentation for its prebiotic effect to be exerted.

The potential prebiotic effect of *T. lutea* partly observed in the fermentation performed in diluted MRS could be related to the fibres contained in the microalgal biomass. About 28% of total dietary fibre remained after digestion, indicating that probably the majority of fibres were soluble and thus easily utilizable. Nevertheless, the fraction left in the digested residue could contribute to prebiotic effects. To further analyse the potential prebiotic effect, trials on a colon resident instead of a lactic acid bacterium could provide more indications. 

### 4.4. Characterization of Nutritional and Functional Properties of Unfermented and Fermented T. lutea F&M-M36

The biochemical composition of *T. lutea* reported in the literature appears rather variable with different macromolecular constituents in turn reported as the most abundant [71,72,73,74,75,76]. In this respect, the data obtained in the present work lie within the range of this variability, with protein being the major constituent followed by lipids and carbohydrates. The fermentation process significantly reduced carbohydrate content, leaving the other main constituents substantially unchanged. To our knowledge, no data on *T. lutea* fermentation are available for comparison. It has to be considered that about one-third of the initial biomass was lost during fermentation, a value much lower than for substrates such as food waste (about 80%) [77], which probably reflects the presence in the microalgal biomass of components difficult to be degraded but also confirms the need for process optimization. This could also partly explain the lack of difference in digestibility of the fermented biomass. As to the digestion residue, its proximate composition is similar to that of raw biomass, i.e., the ratios among different components are almost constant (Table 2). However, if the fraction of each component remaining in the residue is considered, a different picture emerges, as the extent to which each component is conveyed into the fraction that could be considered as bioaccessible changes. In fact, bioaccessibility was 59% for protein, 64% for lipids, and 72% for carbohydrates (Figure 6a). Bonfanti et al. [74] investigated the bioaccessibility of the lipid fraction in a taxonomically close microalga, *Isochrysis galbana*. Although a different *in vitro* digestion protocol was applied, a bioaccessibility of total fats of only about 12% was found. Cavonius et al. [78] evaluated the degree of protein hydrolysis in *Nannochloropsis oculata* using an *in vitro* digestion model (different from that of the present work), finding that 32–50% of the peptide bonds were hydrolysed according to biomass pretreatment. Similar values were obtained by Hori et al. [79] who found a protein digestibility of 43% for *N. commune* biomass. 

As to functional properties, the Italian Ministry of Health [80] states that to consider a product a probiotic, it must contain at least 10^9^ living bacteria in a daily ration based on the assumption that this is the minimum number necessary to permit temporary colonization of the intestine. To exert a probiotic effect, thus, a product based on *T. lutea* F&M-M36 biomass fermented for 72 h should be ingested at a daily ration of 8.4 mL for MRS 1:3 matrix and 21.5 mL for water matrix (2.3 mL if the product is fermented only for 48 h). However, for the probiotic effect to be exerted, it is necessary that the probiotic bacterium survives the passage through the stomach and the proximal part of the intestine. In this respect, the survivability to the simulated stomach passage of *L. plantarum* ATCC 8014 at the end of the fermentation with *T. lutea* was not improved compared to the survivability of the bacterium alone, indicating that microalgal biomass does not have a protective effect, independently of the fermentation matrix. Moreover, a strongly decreased bacterial survivability was observed after the simulation of the intestine passage for both fermentation matrixes. Further investigations are needed to fully clarify these findings. No data are available to our knowledge on the effects on bacterial survivability to *in vitro* digestion after fermentation of microalgal substrates. The addition of unfermented fresh biomass from several microalgae (*C. vulgaris*, *Scenedesmus quadricauda*, *Lagerheimia longiseta* (*L. longiseta*), *S. platensis*) to living probiotic bacteria (*L. acidophilus* 05 or *L. casei* 01), then lyophilized and digested *in vitro*, resulted in not being effective in protecting the probiotic bacteria in the passage through the stomach, but *C. vulgaris*, *L. longiseta*, and *S. platensis* were able to improve survivability after the intestine phase compared to the control in a saline solution [81]. 

One of the most important functional properties of microalgae is antioxidant activity, primarily related to the high pigment content. In this work, radical scavenging activity determined by the DPPH assay was higher in the lipophilic extracts rich in chlorophyll and carotenoids. The activity in the hexane extract containing mainly chlorophyll *a* was low except with high extract concentrations, thus, this molecule is probably not the main antioxidant compound. The activity seems due overall to carotenoids, including fucoxanthin. Fermentation negatively affected total carotenoid content (−36%) and particularly fucoxanthin (−68%). However, fermentation seems to play a positive role by increasing access to pigments, and thus extraction yield, as can be inferred by the increased carotenoid content in both the fermented raw biomass and post-digestion residue. The higher carotenoid content is reflected in the increased radical scavenging activity of the extracts from biomasses fermented in diluted MRS. Increased pigment (carotenoid) extractability could not fully explain the increased antioxidant activity, which might be partly due to the presence of other compounds of microalgal or bacterial origin. Silva et al. [82], for an ethyl acetate extract from the biomass of a commercial *T. lutea*, obtained an IC_50_ of 0.8 g L^−1^, more than half that for lipophilic extracts in this work (about 1.7–1.8 g L^−1^). A water extract from *Pavlova lutheri* KMCC H-006 fermented with the yeast *Hansenula polymorpha* showed an IC_50_ value of 0.3 g L^−1^ [83], an activity much higher than that of the extract in 30% EtOH in water obtained from fermented biomass in the present work, which did not reach values exceeding 50%, making it impossible to calculate IC_50_. It is noteworthy that this extract was the final step of solvent extractions in succession; the lipophilic extracts obtained in the first extraction steps reached IC_50_ values in the range of 0.8–1.0 g L^−1^.

Besides functional properties, to develop products based on fermented *T. lutea* it will be necessary to consider organoleptic features to obtain a palatable product. *T. lutea* biomass is brown, which makes it look unappetizing. Currently, a strategy applied to surpass the colour limit for consumers is to choose shakes, juices, and fruit-based beverages as foods to which to add the microalga since their vivid colours are associated with healthy foods, and they might smooth down the visive impact with the colour of the microalga [12]. However, *T. lutea* has also a strong smell (and taste); a possible strategy would be to choose, as matrix, a sauce/condiment used to season fish, crustaceans, or other marine-derived food. It is remarkable that, as a first impression in the present work, the fermentation process has smoothed the smell of *T. lutea* biomass. This aspect deserves to be investigated with specific sensory analysis because if the first impression will be confirmed, fermentation could represent a good strategy to overcome this limit for *T. lutea* biomass application in foods.

## 5. Conclusions

*T. lutea* F&M-M36 biomass shows a good nutritional profile and rather good digestibility. Bioaccessibility is higher for carbohydrates and, among functional components, for fucoxanthin. *T. lutea* F&M-M36 has a good potential as a substrate for fermentation with *L. plantarum* ATCC 8014, although thorough strain selection for the fermenting probiotic bacterium is recommended for future developments because of the salinity of this marine microalga. Fermentation reduces carbohydrates as well as fucoxanthin. In spite of this, the higher radical scavenging activity in the extracts from biomass fermented in a diluted organic medium suggests that fermentation could make pigments more easily accessible. The role of the fermentation matrix needs further investigation. The presence of the microalgal biomass does not increase the survivability of the probiotic bacterium to the digestive process in the fermented product. Moreover, fermentation does not improve microalgal biomass digestibility. Although some ability of the microalgal post-digestion residue to improve bacterial growth during fermentation was observed, further tests are needed using colon-dwelling bacteria to confirm prebiotic activity, while the growth of *L. plantarum* with *T. lutea* raw biomass seems related to bioaccessible nutritional elements. Finally, in-depth studies will be needed to improve the organoleptic characteristics of this biomass if aiming at wide use in the functional food industry. Approval of *T. lutea* as food by competent authorities would help increase research interest in its application in the food industry, including the development of functional fermented products. 

## Figures and Tables

**Table 1:** Growth rates and doubling times of the three lactic acid bacteria tested. Data were obtained through OD_600_ and cell counts with a Neubauer chamber.

	OD_600_	Neubauer Chamber
Bacterial Strain	Growth Rate (h^−1^)	Growth Rate (h^−1^)
*L. plantarum* ATCC 8014	0.33	0.48
*L*. *bulgaricus* LB28A	0.24	0.33
*L. casei* LB28B	0.17	0.21

**Table 2:** Biochemical composition of *T. lutea* F&M-M36 biomasses and digested residues. Content in protein, carbohydrate, lipid, and total dietary fibres of raw biomass and post-digestion residue, unfermented and fermented. Data are expressed as mean ± standard error. Superscript letters indicate significant differences (*p* < 0.05) between unfermented and fermented samples within the same starting material. TDF, total dietary fibres; nd, not determined.

	Protein	Carbohydrate	Lipid	Ash	TDF	Total Carotenoids	Fucoxanthin
	% (Dry Weight)					mg g^–1^ (Dry Weight)	
Raw biomass							
unfermented	42.0 ± 2.5	12.6 ± 0.7 ^a^	29.3 ± 0.3	11.9	9.3	20.8 ± 0.3 ^a^	5.86 ± 0.20 ^a^
fermented in H_2_O	48.6 ± 4.0	7.2 ± 0.5 ^b^	32.6 ± 5.7	9.1	nd	12.3 ± 1.5 ^b^	2.00 ± 0.16 ^b^
fermented in MRS 1:3	48.9 ± 1.1	7.9 ± 0.4 ^b^	33.4 ± 4.4	6.2	nd	13.3 ± 0.3 ^b^	1.88 ± 0.03 ^b^
Post-digestion residue							
unfermented	45.9 ± 4.1	10.2 ± 0.4 ^a^	30.6 ± 0.3	10.7	7.4 ± 1.3	11.3 ± 1.1	1.26 ± 0.01
fermented in H_2_O	43.8 ± 1.0	8.8 ± 0.8 ^b^	33.4 ± 3.4	5.8	nd	11.2 ± 0.4	1.19 ± 0.00
fermented in MRS 1:3	39.8 ± 1.6	8.3 ± 0.1 ^b^	32.9 ± 6.6	5.7	nd	11.4 ± 0.0	0.85 ± 0.08

## Data Availability

The data presented in this study are all available within the article.
